# Efficacy of educational interventions in adolescent population with feeding and eating disorders: a systematic review

**DOI:** 10.1007/s40519-023-01594-9

**Published:** 2023-08-22

**Authors:** Sergio Ladrón-Arana, Rosario Orzanco-Garralda, Paula Escalada-Hernández, Carlos Aguilera-Serrano, Marta Gutiérrez-Valencia, Jordi Urbiola-Castillo

**Affiliations:** 1 Mental Health Management, Navarre Health Service, Pamplona, Spain; 2 Department of Health Sciences, Public University of Navarre (UPNA), Avda. Barañain, S/N-31008, Pamplona, Navarre Spain; 3 Navarra Institute for Health Research (IdisNA), Pamplona, Spain; 4 Mental Health Clinical Management Unit, Regional University Hospital, Malaga, Spain; 5 Unit of Innovation and Organization, Navarre Health Service, Pamplona, Navarre Spain; 6 Mental Health Intermediate-Term Unit, Navarre Health Service, Pamplona, Spain

**Keywords:** Adolescent, Children, Feeding and eating disorders, Health education

## Abstract

**Background:**

Educational interventions are a key element in the care of young patients with feeding and eating disorders, forming part of the majority of therapeutic approaches. The aim of this review is to evaluate the impact of educational interventions in adolescents with feeding and eating disorders.

**Methods:**

Following the PRISMA recommendations electronic databases were searched up to 29 June 2023. Studies related to educational interventions in young population diagnosed with feeding and eating disorders (anorexia nervosa, avoidant/restrictive food intake disorder, bulimia nervosa, pica and ruminative disorders and binge- eating disorder) in Spanish and English language, without temporal limitation, were located in the databases: PubMed, Scopus, CINAHL, Cochrane Library, PsycINFO, CUIDEN, DIALNET, and ENFISPO. A search in the databases of grey literature was performed in OpenGrey and Teseo. The review protocol was registered in PROSPERO (CRD42020167736).

**Results:**

A total of 191 articles were selected from the 9744 citations screened. Ten publications were included. The results indicated variability between educational programs, including individual and group interventions, learning techniques and various research methodologies. Variables such as learning, attitudinal and perceptual changes, anthropometric parameters, symptom improvement, normalization of eating patterns, evaluation of the program and cognitive flexibility were identified. The risk of bias was high due to the low methodological quality of a large number of studies analyzed.

**Conclusion:**

The results indicate that educational interventions can influence the improvement of knowledge level and have a positive effect on health outcomes. Although education is a common practice in the treatment of these pathologies, high-quality studies were not identified. Thus, this review concludes that additional evidence is needed to evaluate the effectiveness of educational programs, with further research studies, especially randomized controlled trials, to confirm these results.

**Level of evidence:**

Level I: Systematic review.

**Supplementary Information:**

The online version contains supplementary material available at 10.1007/s40519-023-01594-9.

## Introduction

Feeding and eating disorders (FEDs) are a group that includes a variety of diagnoses including anorexia nervosa, avoidant/restrictive food intake disorder, bulimia nervosa, pica and ruminative disorders and binge-eating disorder.

Although some of these pathologies share common elements, the diagnostic criteria for each of them are particular. Table 1 summarizes the most important diagnostic features according to the DSM-V criteria. [1]

**Table 1:** Feeding and eating disorders diagnostic criteria

Disorder	Diagnostic criteria
Anorexia nervosa	• Food intake restriction • Significantly low body weight in relation to age and sex • Intense fear of weight gain • Body image distortion
Avoidant/restrictive food intake disorder	• Significant weight loss • Significant nutritional deficiency • Interference with psychosocial functioning • Not explained by lack of food or culturally accepted practice • It does not occur in the course of another eating disorder or medical condition
Bulimia nervosa	• Recurrent binge eating at least once a week • Compensatory behaviors (self-induced vomiting, use of laxatives, fasting, excessive exercise, etc.) at least once a week • May not have alterations in nutritional values
Pica	• Persistent ingestion of non-food substances for a minimum period of one month outside the culturally and socially accepted context
Ruminative disorder	• Repeated regurgitation for a minimum period of one month not associated with another medical condition or eating disorder
Binge-eating disorder	• Recurrent binge eating (at least once a week for three months) not associated with inappropriate compensatory behavior outside of the course of bulimia nervosa

The onset of FEDs occurs mainly at early ages, with 18 years being the mean age for diagnosis of anorexia nervosa (AN) and bulimia nervosa (BN) [2]. It has been shown that the prognosis for detecting EDs is more favorable in adolescents than in adults, so early care should be applied. When FEDs are diagnosed in adolescence, several studies have estimated that up to 57% of patients recovered, 25.95% improved and 16.9% had a chronic course [3].

The available evidence confirms that due to the complex individual and environmental characteristics of each patient and family, together with the high rate of comorbidity (psychiatric and medical), a multidisciplinary approach is necessary, involving the combination of several therapeutic strategies [4–6]. However, there is not enough evidence to affirm that a specific therapy provides more benefits than another [5–8]. Therefore, treatment must be adapted taking into account the particularities of the patient and the environment.

Nutritional rehabilitation is a common element of many evidence-based treatments for FEDs [9]. Among the therapeutic strategies, psychological interventions are considered as an effective option for treating FED’s [10], including cognitive behavioral therapy (CBT). Nevertheless, specific types of psychological interventions may be effective for specific populations like family therapy for children and young people with AN [11].

Educational interventions (EIs) are one of the key elements in the treatment for patients with FED’s. EIs are a tool used in a variety of therapeutic approaches for patients with feeding and eating disorders. Unlike therapeutic approaches, which are systematic and structured, EIs are specific and concrete strategies that are used as part of a therapeutic approach to provide information and teach skills to patients [12]. EIs can include informative lectures, group discussions, individualized counselling, relaxation exercises, use of new technologies, and other teaching approaches that help patients better understand their disorder and develop skills to manage it effectively These usually are applied in combination with other treatment modalities. Therefore, there is not much evidence that particularly assesses the impact that these interventions have on this population [12].

Despite being a term with multiple meanings, EIs could be defined as “those consciously planned interventions that try to communicate information (verbal, written or audiovisual) to individuals, groups or communities, with the aim of improving their knowledge and skills, to allow them to make correct health decisions” [13]. There is some experience that shows how this type of interventions can be useful in the preventive field [14], favoring the understanding of the disease, learning self-care skills, improving health status, reducing the use of health care and minimizing the general burden of the condition [15, 16].

The educational strategies and approaches used in patients diagnosed with EDs are varied, including both the group education format [17] and the nutritional rehabilitation instruction [18]. EIs are not only useful in increasing knowledge, but also act as a basis or structure for other therapeutic interventions [19].

It is important to consider that implementing educational programs does not guarantee their success and that there is not enough evidence to know the results of these interventions. Therefore, a dynamic evaluation of the program’s development and its impact might be necessary to identify the health objectives achieved and their effectiveness. However, the studies on the different interventions applying educational strategies for patients with FEDs have not been systematically reviewed to offer healthcare professionals involved in their care the best evidence to guide their practice. Considering the importance of early care in adolescents with FEDs to ensure a good prognosis, it is important to analyze the evidence on educational interventions as a therapeutic tool in this population.

### Aims

The aim of this review is to synthesize the best available evidence on the effectiveness of EIs, in combination or not with other treatment strategies, in adolescent population with feeding and eating disorders (anorexia nervosa, avoidant/restrictive food intake disorder, bulimia nervosa, pica and ruminative disorders and binge-eating disorder).

## Methods

A systematic review was carried out following the PRISMA recommendations to address the objective of the work and for the selection and representation of studies [20].The review protocol was registered in PROSPERO (registration number CRD42020167736).

### Search strategy and selection criteria

A systematic search of scientific literature was performed on June 29, 2023, in the following databases: PubMed (MEDLINE), Scopus, CINAHL (Cumulative Index to Nursing and Allied Health Literature), Cochrane Library (CENTRAL, Cochrane Central Register of Controlled Trials), PsycINFO, CUIDEN, DIALNET, and ENFISPO. Likewise, a search was carried out in the grey literature databases OpenGrey and Teseo to detect unpublished studies. It was limited to studies in Spanish and English, with no country or temporal restriction by year of publication, since no reviews were found covering previous time periods.

The search was designed with Medical Subject Headings (MeSH) terms for MEDLINE and was adapted for the rest of the databases according to their descriptors or using keywords. The following terms were combined: “adolescent”, “eating disorder” and “educational intervention”. The complete search strategy is provided in the supplmentary file 1.

Once the duplicates were eliminated, the screening process was undertaken independently by two reviewers (SL and JU) using a four-stage blinded approach: (I) review of titles and abstracts; (II) examination of full texts with respect to inclusion and exclusion criteria; and (III) screening the reference lists of all included and in-scope studies to identify further eligible studies. Discrepancies were resolved by consensus, and although the research protocol specified that in the absence of consensus among the reviewers, a third party (CS) would be consulted, it was not necessary. For these purposes, the screening web-tool system Covidence was used [21].

### Inclusion and exclusion criteria

#### Types of studies

Studies, with experimental or quasi-experimental designs, that evaluated the effectiveness of educational interventions, in combination or not with other treatment strategies, in patients with FEDs.

#### Participants

Studies that involved populations aged 12 to 18 of both sexes with a diagnosis of FEDs as defined by the DSM-5 criteria [1] or previous versions, which include the diagnoses of anorexia nervosa, avoidant/restrictive food intake disorder, bulimia nervosa, pica, ruminative disorders and binge-eating disorder. Studies with broader age ranges that included data in the range of interest were also considered depending on their specific analysis and if data on the population of interest could be disaggregated.

Studies were selected if they carried out educational interventions according to the WHO definition: “those consciously planned interventions that try to communicate information (verbal, written or audiovisual) to individuals, groups, or communities, with the aim of improving their knowledge and skills to allow them to make correct health decisions” [13] or other comparable strategies. We included studies that applied group or individual interventions in healthcare setting.

Studies conducted in patients with anorexia as a symptom, defined as lack of appetite or appetite affected by multiple causes, such as organic anorexia that originates due to an underlying disease, were excluded [22]. In addition, studies were excluded if the interventions aimed exclusively at families or support groups.

#### Outcome variables

Studies that analyzed the effectiveness of the interventions were eligible. Those that included at least one of the outcome variables represented in Table 2 were selected.

**Table 2:** Outcome variables

Primaries	Secondary
Knowledge acquired	Satisfaction
Decrease erroneous beliefs	Anxiety
Symptom improvement	Depression
Changes in behavior/life habits	Motivation
BMI	Therapeutic adherence
Decrease compensatory behaviors	Hospital admissions and readmissions
	Other variables of interest

### Study selection and data extraction

Two reviewers (SL and JU) independently extracted the relevant data using the JBI-SUMARI tool for quasi-experimental studies [23] and JBI-SUMARI tool for randomized controlled trials [24].

The data studies were extracted into a standardized table that included: author’s name, year of publication, country, type of educational program, group or individual approach, study design, sample size, gender, program duration, outcome variables, measurement tools, and main results, as shown in Table 3.

**Table 3:** General characteristics of the included studies

Article	Country	Program	Design	Size/gender	Duration	Variable	Scale	Results				Conclusion	
								Pre	Post	Pre	Post		
Geist et al. (2000)	Canada	Group psychoeducation of families	Quasi-experimental	*N* = 25 Female	4 Months	BMI (weight gain)	% Weight gain	74.9%	91.3%	77.2%	96.3%	Significant improvement in both interventions	
						Improvement in symptoms Changes in behavior and/or lifestyle	EDI-2 Eating Disorder Inventory (Symptoms)	Drive for thinness11.1 Body image satisfaction 9.1 Improved bulimia symptoms 1.2	Drive for thinness12.3 Body image satisfaction 10.6 Improved bulimia symptoms 1.2	Drive for thinness 13.7 Body image satisfaction Pre 11.0 Improved bulimia symptoms 1.9	Drive for thinness 13.3 Body image satisfaction Pre 12.2 Improved bulimia symptoms 2.5	No significant difference in both groups	
							BSI (Brief Symptom Inventory)	Patient 1.3 Mother 0.7 Father 0.7	Patient 1.2 Mother 0.6 Father 0.4	Patient 1.4 Mother 0.6 Father 0.4	Patient 1.2 Mother 0.6 Father 0.3	No significant difference in both groups	
						Depression	CDI (Children Depression Inventory)	11.8	12.2	14	15.4	No significant difference in both groups	
						Family functioning	FAM-III (Family Assessment Measure)	48.3	52.2	50.9	55.8	Significant improvement in both interventions appreciate the presence of relatives in both groups	
						Average stay of admission	Days stay	46.3		40.8		No significant difference in both groups	

Article	Country	Program	Design	Size/gender	Duration	Variable	Scale	Results				Conclusions	
								Diet		Control			
								Pre	Post	Pre	Post		
Andrewes et al. (1996)	Australia	Program-Based Group Computerized Intervention DIET Freq: NR Dur: NR LOI: NR Set: Hospital	Quasi-experimental with control group	*N* = 54 Female 27 Diet 27Control	No specific	Improvement in symptoms Changes in behavior and/or lifestyle	Eating disorders attitude questionnaire	149	166,9	148,6	149,4	Significantly greater positive change in the DIET group versus the control	
							Eating disorders knowledge questionnaire	30,1	44,4	34,4	37,1	Significantly greater increase in knowledge in the DIET group compared to the control	

Article	Country	Program	Design	Size/gender	Duration	Variable	Scale	Results				Conclusions	
								Pre	Post				
Loria Kohen et al. (2009)	Spain	Individual nutrition education program Freq: 1/week or 1/15 days Duration: 15–20 sessions LOI: 4–6 months Set: Consultation	Quasi-experimental with pre–post measurements	*N* = 89 5% Men’s 95% Women	4–6 months	Average questionnaire value	EAT 26	32 ± 15	23.7 ± 14			Therapeutic changes significant improvement	
						Diet	EAT 26	16.7 ± 9	13 ± 9			Significant improvement	
						Concern about food	EAT 26	7.1 ± 5	5.1 ± 4			Significant improvement	
						Oral control	EAT 26	7.5 ± 5	5.8 ± 5			Significant improvement	
						Number of meals per day	Normal pattern 4 daily meals (Dietary survey)	70% less than 4 meals a day	19% less than 4 meals a day			Significant improvement	
						Meal time	Normal pattern 26–44 min (Dietary survey)	46% patients within normal	67% patients within normal			Significant improvement	
						Full meal intake	Normal pattern 1st, 2nd dish, bread and dessert (Dietary survey)	30% patients full meal	54% patients full meal			Significant improvement	
						Compensatory behaviors	Nº vomits per week Binge week Excessive water intake Excessive physical activity (≥ 2 h daily)	ANP(7.2 ± 10) BN (8 ± 9.7) TANE (1.6) ANP (2.3) BN (6.5) TANE (1.8) 10% 16%	ANP(1 ± 1.8) BN (2.2 ± 3.2) TANE (0.7) ANP (0.25) B.N (2.1) TANE (0.9) 1.5% 3%			Significant improvement in all three diagnoses	
						Changes in energy intake	(Dietary survey)	ANP 960 ± 600 kcal ANR 1120 ± 400 BN not measurable	ANP 1290 ± 500 kcal ANR 1545 ± 400 BN not measurable			Improvement in the two BN diagnoses not applicable due to binge eating	
						BMI	BMI calculation	ANR 17.6 ± 2.2 ANP 19.4 ± 2.4 TANE 21.1 ± 3.2 B.N 26.4 ± 6.6	ANR 18.9 ± 2.2 ANP 19.8 ± 2.1 TANE 21.3 ± 4 B.N 25.5 ± 5.7			Significant improvement in ANR ANP and TANE remained normal B.N Evolution towards normalization	
						Evolution of consumption by food groups	Range recommended by SENC (Spanish Society of Community Nutrition) (Dietary survey)	Dairy 61% out of range Vegetables 69% out of range Fruits 84% ​​out of range Cereals 90% out of range Meats 58% out of range Oil 45% out of range	Dairy 82% within range Vegetables 70% within range Fruits 33% within range Cereals 39% within range Meats 52% within range Oil 75% within range			Dairy, vegetables, cereals and oil, highly significant improvement Fruits, meats significant improvement	

Article	Country	Program	Design	Size/gender	Duration	Variable	Scale	Results				Conclusion	
								Group CRT (Cognitive remediation Therapy)		Group HC (Healthy controls)			
								Pre	Post	Pre	Post		
Van Noort et al. (2016)	Germany	CRT group(Cognitive Remediation Therapy)	Quasi-experimental with control group	*N* = 40 Female 20 CRT 20 Healthy group	5–10 Weeks	Anxiety	STAI	64.8 ± 11.8		41.0 ± 6.3			
						Depression	German depression inventory for children and adolescents	3.6 ± 1.7		0.6 ± 0.7			
						Cognitive flexibility Learning effects	TMT-4	55.9 ± 13.2	51.7 ± 13.6	47.3 ± 9.7	50.2 ± 12.3	Significant improvement in both groups in the CRT group	
						Central coherence Learning effects	RCFT	1.05 ± 0.43	1.31 ± 0.31	1.03 ± 0.41	1.17 ± 0.51	No significant differences	
						Satisfaction	FBB-P		3.1 ± 0.4		2.6 ± 0.6	Improvement	
							Piers–Harris Children’s Self-Concept Scale (PHC-SCS), the Self- Esteem in Eating Disorders Questionnaire (SEED) and Socialization Battery (BAS-3)						

Article	Country	Program	Design	Size/gender	Duration	Variable	Scale	Results				Conclusion	
								Pre		Post			
Lazaro et al. (2011)	Spain	Cognitive group program of techniques to improve and maintain self-esteem	Quasi-experimental with pre–post measurements	*N* = 160	4 months	BMI	Mean	AN-rd = 18.7 BN-rd = 21.1		AN-rd = 19.6 BN-rd = 21.4		Significant differences in both groups (AN-rd y BN-rd)	
						Self-esteem	PHC-SCS Behavior adjustment Intellectual/school status Physical appearance Freedom for anxiety Popularity Happiness/satisfaction	AN-rd 9.7 (3.3) 8.8 (4.2) 3.6 (3.2) 3.7 (2.9) 5.9 (3.2) 2.1 (4.5)	BN-rd 8.4 (3.9) 7.2 (4.3) 3.2 (2.8) 3.1 (2.4) 6.2 (2.7) 1.5 (1.8)	AN-rd 9.5 (3.7) 8.8 (4.5) 4.3 (3.6) 4.2 (3.3) 6.1 (3.4) 2.7 (2.7)	BN-rd 9.1 (3.7) 8.9 (3.9) 5.2 (3.5) 4.7 (3.1) 7.0 (3.1) 3.0 (2.4)	Both groups (AN-rd and BN-rd) showed significant improvements in their perceptions of physical appearance, their weight-related self-concept	
						Self-esteem	SEED SC in relation to others SC related	AN-rd 16.5 (9.7) 14.6 (7.8)	BN-rd 17.3 (7.8) 17.6 (7.0)	AN-rd 15.0 (10.7) 13.5 (9.0)	BN-rd 13.2 (8.5) 13.2 (8.0)	Both groups (AN-rd and BN-rd) presented significant improvements in the perception of the form of relationship with others	
						Social skills	BAS-3 Consideration for others Self-control in social relations Social withdrawal Social anxiety/shyness Leadership	AN-rd 58.3 (37.5) 51.4 (31.5) 77.7 (24.1) 68.2 (29.3) 39.1 (34.0)	BN-rd 52.5 (39.0) 40.6 (27.5) 82.6 (21.3) 67.8 (33.3) 35.5 (31.9)	AN-rd 56.8 (37.9) 50.6 (32.8) 73.4 (25.0) 67.1 (32.3) 43.8 (36.6)	BN-rd 63.6 (36.5) 45.5 (31.7) 69.5 (26.1) 59.9 (34.7) 51.8 (36.4)	Both groups (AN-rd and BN-rd) showed significant improvements in their perceptions about social isolation and leadership	

Article	Country	Program	Design	Size/gender	Duration	Variable	Scale	Results					Conclusions
								Pre	Post	3 months	6 months	1 year	
Johnston et al. (2015)	United States	Skillstreaming the Adolescent by Goldstein and McGinnis (1997) DBT group	Quasi-experimental with pre–post measurements	*N* = 51	4 months	BMI	Mean global BMI (kg/m^2^) (*n* = 36)	17.373 ± 2.010	18.319 ± 1.764	18.895 ± 1.890	19.087 ± 1.906	19.705 ± 1.740	Improvement in weight gain over weight
						Menstruation status	% had regular menses (based on parent report)			53%	61%	78%	Improved regulation of the menstrual cycle
						Psychological measures	Mean global EDE-Q (*n* = 33)	3.15 ± 1.52	2.11 ± 1.49	we’re not gathered at this follow-up	(*n* = 19) 1.64 ± 1.17	(*n* = 16) 1.59 ± 1.54	Psychological improvement in thoughts, attitudes and behaviors of eating disorders No significant differences were found for binge frequency, F (4, 92) = 1.643, p = .17, or purge frequency, F(4, 92) = 1.626, p = .17, over the course of treatment or the subsequent year

Article	Country	Program	Design	Size/gender	Duration	Variable	Scale	Results			Conclusion		
								Baseline	3 months	1 year			
Ruiz Prieto et al. (2013)	Spain	Psychological improvement in thoughts, attitudes and behaviors of eating disorders	Quasi-experimental with pre–post measurements	*N* = 106	1 Year	Quality of diet choice	Caloric content	1868.93	2062.22	2010.22	Improvement in the time they spent choosing the diet, the BMI was normalized and they chose qualitatively better diets, with higher carbohydrate content although the fat content was maintained		
							Fat content	37.75g	43.22				
							Carbohydrate content	203.7	224.8				
						Time spent on diet choice		20, 48 min	17.67 min	13.48 min			
						BMI		20.92	21.95	22.10			
						Body Fat Mass		19.48%	21.53%	21.72%			

Article	Country	Program	Design	Size/gender	Duration	Variable	Scale	Results			Conclusion		
								Pre	Post	3 months			
Sternheim et al. (2018)	Australia	Group cognitive behavioral program with psychoeducational sessions	Quasi-experimental with pre–post measurements	*N* = 10 Women	3 Months		Depression	BDI	20.5		Statistically significant reduction in IU in patients with anxious and depressive symptomatology		
							Anxiety	State anxiety Percentile	85.5				
								Trait anxiety Percentile	82				
							Acceptability	Patient satisfaction questionnaire		7.03			
							Feasibility	Discussion in the service		Yes			
							Intolerance of uncertainty	Intolerance of Uncertainty Scale (IUS)	94.8	76.6	71.4		

Article	Country	Program	Design	Size/gender	Duration	Variable	Scale	Results				Conclusion	
								Group CRT (Cognitive remediation Therapy)		Group NSCT			
								Pre	Post	Pre	Post		
(Herbrich‐Bowe et al*.*, 2022)	Germany	CRT individual(Cognitive Remediation Therapy) Vs NSCT (Non-specific cognitive training)	Randomized controlled trial	*N* = 56 Female 28 CRT Vs 28 NSCT	5 Weeks (10 sessions)	Cognitive flexibility Learning effects	WSCT	2.5 ± 0.7	2.5 ± 1.2	1.7 ± 0.7	2.3 ± 1.1	No significant differences. Both groups improved over time	
						Central coherence Learning effects	GEFT	13.6 ± 0.8	15.3 ± 0.9	12.5 ± 0.9	13.4 ± 1.0	No significant differences. Both groups improved over time	
						Cognitive flexibility Learning effects	TMT-4	11.6 ± 0.5	11.2 ± 0.6	11.5 ± 0.4	11.8 ± 0.5	No significant differences. Both groups improved over time	
						Central coherence Learning effects	CCI	1.2 ± 0.1	1.3 ± 0.8	1.4 ± 0.1	1.3 ± 0.8	No significant differences. Both groups improved over time	
						Every day-life flexibility Task completion	BRIEF-SR	45.1 ± 1.9	45.8 ± 2.1	48.1 ± 2.0	44.8 ± 2.1	No significant differences. Both groups improved over time	

Article	Country	Program	Design	Size/gender	Duration	Variable	Scale	Results				Conclusion	
								Group MCT-ED		Group TAU			
								Post-intervention	3-month follow-up	Post-intervention	3-month follow-up		
(Balzan et al*.*, 2023)	Australia	MCT-ED individual(Metacognitive training for eating disorders) Vs TAU (Treatment as usual)	Randomized controlled trial	*N* = 35 Female 20 MCT-ED Vs 15 TAU	6 Weeks (6 sessions)	Perfectionism: personal standards	The Frost Multidimensional Perfectionism Scale	25.84(.78)	25.36(.91)	27.68(.86)	27.35(1.13)	No significant differences No significant reductions over follow-up	
						Perfectionism: concern over mistakes	The Frost Multidimensional Perfectionism Scale	28.43(1.30)	30.62(1.29)	35.12(1.54)	34.67(1.63)	Short-term reductions, with large effect size, although a significant difference between groups was not observed at the follow-up	
						Eating disorder pathology	Eating Disorder-15	3.90(.24)	3.27(.30)	4.10(.29)	3.77(.37)	No significant differences No significant reductions over follow-up	
						Body image flexibility	Body Image Acceptance and Action Questionnaire	35.08(2.77)	37.51(3.67)	34.33(3.44)	35.18(4.57)	No significant differences No significant reductions over follow-up	

## Results

**Fig. 1:**
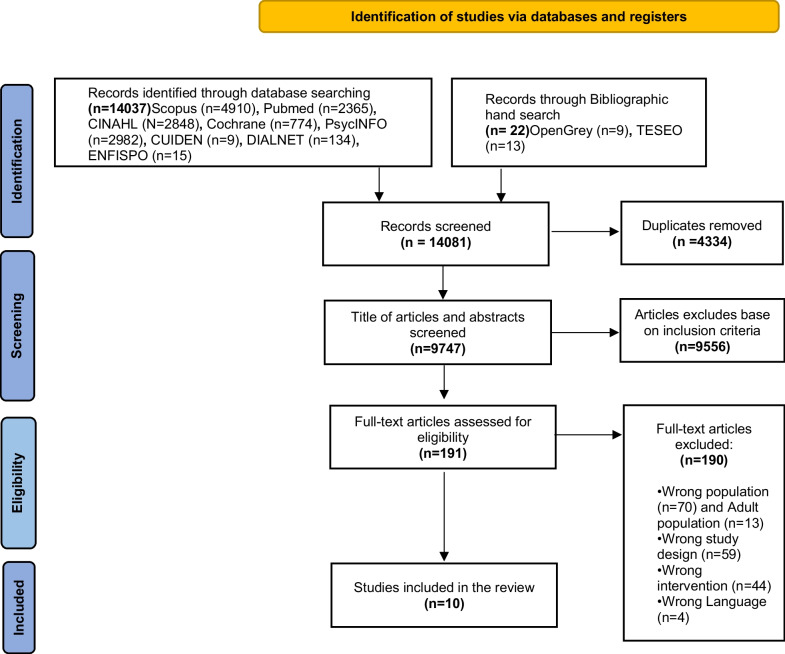
PRISMA 2020 flow diagram

Figure 1 illustrates the selection process using the PRISMA flowchart.

A total of 9747 potentially relevant studies were identified after excluding duplicates. After title and abstract screening, 191 studies were assessed as potentially meeting the inclusion criteria. Full text review resulted in nine  studies meeting the inclusion criteria, and the reference lists of the included studies yielded one additional paper. A total of ten studies were included in our review.

For the execution of this procedure, the screening web-tool system Covidence [21] was used.

### Characteristics of included studies

The ten studies finally included in the review were conducted in five countries, three in Spain, two in Australia, Canada and Germany and one in the United States.

The studies evaluated different educational methodologies and assessed their effects using different tools, so comparisons were difficult to make.

Although we only found two randomized controlled trials, three studies had a control group [25–27]. In the work of Andrewes et al. [25], two independent interventions were measured by performing prior and subsequent controls on both groups and comparing them. Van Noort et al. [27] performed a pre–post test evaluation in which an intervention group was compared with a healthy control. In the case of recent studies [28, 29], two different interventions were compared in order to analyze which was more effective.

Regarding the remaining five studies, that lacked a control group [30–32], presented a quasi-experimental design in which the results obtained pre- and post-intervention were measured. Quasi-experimental studies were also used in other two studies [33, 34], but in addition to measuring the pre- and post-intervention results, successive measurements were performed while the interventions were being developed.

In the selected studies, a total of 626 patients participated, of which 516 completed the respective studies; in three studies, the existence of possible dropouts was not specified [25, 30, 31]. The sociodemographic characteristics of the participants in the selected studies were similar. The vast majority of the participants were female, and only in one study [31] was the inclusion of four male participants specified. The samples of most of the studies were within the age range proposed in the review protocol (12–18 years). Three studies included higher age ranges [26, 30, 31], but these were extreme values of the distribution, so the average age remained low.

The studies analyzed different types of pathologies included within feeding and eating disorders. In several of them, subtypes were specified, such as unspecified AN [26–29, 32], or both AN and BN [25, 30, 33].

Regarding the variables analyzed, as seen in Table 3, knowledge acquired was assessed in all the selected studies using different knowledge tests[26] or indirectly by assessing changes in behavior or participants’ dietary patterns[31]. In addition, variables such as cognitive flexibility [27–29], learning social skills [31], perfectionism [29] attitudes and thoughts towards food [34], the quality of the choice of diet and the time it took to prepare it [33], or tolerance of uncertainty [32] were studied. Other variables evaluated in several studies highlighted the improvement in symptoms, a decrease in anxiety, and improvement in depression or changes in anthropometric parameters, self-esteem, family functioning, and average stay of admission. A summary of these variables and measurement instruments is shown in Table 3.

### Risk of bias

The selected articles were evaluated by two reviewers (SL and JU) independently based on methodological validity and risk of bias, according to the criteria developed by JBI Critical Appraisal Tools [24] in order to ensure their rigor and quality. In the event of a disagreement between the reviewers, consensus prevailed after discussion, and it was not necessary to consult a third reviewer.

Across the ten studies included in this review, three studies had a low risk of bias across all domains, while seven studies had a low risk of bias in at least two out of the eight domains. No studies presented a high risk of bias in all domains; however, four studies had a high risk of bias in at least three domains. A summary of the risk of bias assessment is presented in Tables 4 and 5.

**Table 4:** Methodological quality assessment

JBI critical appraisal checklist for quasi-experimental studies									
Article	Is it clear in the study what is the cause’ and what is the ‘effect’	Were the participants included in any comparisons similar?	Were the participants included in any comparisons receiving similar treatment/care, other than the exposure or intervention of interest?	Was there a control group?	Were there multiple measurements of the outcome both pre and post the intervention/exposure?	Was follow-up complete and if not, were differences between groups in terms of their follow-up adequately described and analyzed?	Were the outcomes of participants included in any comparisons measured in the same way?	Were outcomes measured in a reliable way?	Was appropriate statistical analysis used?
Geist, R et al. 2000 Canada	O	O	O	O	O	O	O	O	O
Andrewes, et al. 1996. Australia	O	O	O	O	O	O	O	O	O
Loria Kohen, et al. 2009 Spain	O	X	X	X	O	O	*	O	O
Van Noort, et al. 2016 Germany	O	O	O	O	O	O	O	O	O
Lázaro, L., et al. 2011 Spain	O	O	X	X	O	O	O	O	O
Johnston, JA.et al., 2015 United States	O	O	X	X	O	O	*	*	O
Ruiz-Prieto, et al. 2013. Spain	O	X	X	X	O	O	O	O	O
Sternheim, L.et al. 2018 Australia	O	X	X	X	O	O	O	O	O

Yes: O; No: X; Unclear:*

**Table 5:** Methodological quality assessment

JBI critical appraisal checklist for randomized controlled trials												
Article	Was true randomization used for assignment of participants to treatment groups?	Was allocation to treatment groups concealed?	Were treatment groups similar at the baseline?	Were participants blind to treatment assignment?	Were those delivering treatment blind to treatment assignment?	Were outcomes assessors blind to treatment assignment?	Were treatment groups treated identically other than the intervention of interest?	Was follow-up complete and if not, were differences between groups in terms of their follow-up adequately described and analyzed?	Were participants analyzed in the groups to which they were randomized?	Were outcomes measured in the same way for treatment groups?	Was appropriate statistical analysis used?	Was the trial design appropriate, and any deviations from the standard RCT design (individual randomization, parallel groups) accounted for in the conduct and analysis of the trial?
(Herbrich‐Bowe et al*.*, 2022)	O	O	O	O	X	*	O	O	O	O	O	O
(Balzan et al. 2023)	O	X	O	*	X	*	O	O	O	O	O	O

Yes: O; No: X; Unclear:*

When interpreting the results of this work, it is important to consider that the effectiveness may vary given the variety in intervention and study types, as well as a high likelihood of publication bias, where studies with positive results are more likely to be published.

Regarding the methodological assessment, the results of the evaluation are summarized in Table 4 for quasi-experimental studies and Table 5 for randomized controlled trials. In all the studies, multiple outcome measurements were performed before and after the interventions, the follow-up was completed, and the differences between the groups were adequately described and measured in the same way (in the studies that had comparison), and the statistical analysis was appropriate. Thus, it can be concluded that there was no confusion between the cause-and-effect variable in any of the studies.

### Characteristics of the educational interventions

Although the interventions evaluated and implemented in the selected studies were heterogeneous in several aspects, in all of them, the theoretical basis, the place where the intervention was developed and its content were described. The interventions were carried out both in the hospital setting [25, 26, 28, 32] and in outpatient [29, 30, 34] or mixed [27] settings, such as Day Hospital in FEDs [31, 33].

The educational activity was conducted in groups in six of the studies [26–28, 30, 32, 33] and one-on-one in four studies [25, 29, 31, 34]. The educational intervention was developed simultaneously with the usual care in most of the studies.

In seven cases, the educational intervention was directed to patients, except in three studies [27, 30, 34] in which the intervention was aimed at both patients and relatives. Interventions were performed once a week [29, 31–33], once every 15 days [26, 30], twice a week [27, 28] or three times per week [34]. One study did not specify the frequency of the sessions [25]. In the studies with a control group, participants received either no intervention or the usual intervention of the service in which they were patients.

In the analyzed studies, the educational interventions were applied as a tool as part of different therapeutic approaches, including group psychoeducational therapy [30–33], family psychoeducational therapy [26], cognitive rehabilitation therapy [27, 28], dialectical behavioral therapy [34] use of computerized audiovisual programs in a group psychoeducational therapy [25] and metacognitive training [29].

Regarding the main results reported on the effectiveness after the interventions, all studies directly or indirectly measured the effects on learning, finding improvements in knowledge in relation to the problem of nutrition [25, 27, 33], and positive attitudinal and perceptual changes towards the object in question, food [25, 31, 32].

In four of the studies [26, 30, 33, 34], weight gain was found after the educational intervention. Some studies also reported significant improvements in relation to the symptomatology characteristic of these disorders, and very particularly, in the decrease in the frequency of vomiting, binge-eating disorder and purging [30, 34].

In the post-intervention follow-up, even when intervals between the different studies differed, the general results show a very favorable trend with respect to the normalization of eating patterns and the maintenance of psychopathological measures [30, 34]. Only two studies [25, 26] compared the effects of two interventions between two groups, but only slightly significant differences were identified between both groups in the pre- and post-intervention measurements. A third comparative study between two groups (i.e., ED group vs. healthy group) that received the same intervention showed improvement in cognitive flexibility in intervention group [27]; however, the lack of a control group does not allow the results to be compared with the commonly used treatment. In the study of Herbrich-Bowe et al. [28], a cognitive training intervention was compared with a non-specific intervention, the results in this case did not show superiority of one intervention over another. Similar case to Bazan et al. [29] who evaluated a metacognitive treatment program with treatment as usual, with a significant group difference post-intervention but not 3-month follow-up.

Six studies recognize that the results should be evaluated considering that the small sample size included in some of the studies makes it difficult to determine significant relationships between the variables [26, 27, 29, 32–34].

## Discussion

The results obtained in this study suggest that educational interventions have the potential to improve the knowledge, skills, attitudes, and behaviors of people with FEDs in relation to their health and can produce improvements in health outcomes. Of the ten studies that were selected according to the inclusion criteria, all but one [28] showed to a greater or lesser extent the impact of education on different variables, including knowledge, anthropometric data and symptom improvement. However, the heterogeneity of both measurement instruments and types of study has been very important.

The countries where the studies included in this work were conducted were Western countries. One possible explanation for this phenomenon is that in these countries, the prevalence rate of eating disorders is higher.[35] Additionally, it is important to consider a potential selection bias when analyzing the findings presented, as the articles included were written in Spanish or English.

Therefore, the conclusions of this review should be interpreted with caution due to some methodological issues. First, the methodological quality of the included studies varied substantially. Although we identified some studies with a rather low methodological quality, we did not exclude them from our review due to the lack of studies that address the chosen topic. We observe that the most recent studies generally have greater methodological rigor. In this regard, the works carried out by Herbrich-Bowe et al. [28] and Van Noort et al. [27] meet all the quality criteria in the methodological evaluation performed; an assessment similar to that obtained by the study performed by Geist et al. [26], which had high methodological rigor but was limited by lack of a control group. In contrast, other researches included [29, 30, 32, 33] did not have such a high methodological rigor.

Second, the types of studies that were included in our review were not homogeneous. Different methodological designs, educational interventions and care devices were evaluated, although the populations studied were comparable.

Education can be conducted individually or with groups, and this choice depends on the patient's needs at any given moment. Both strategies have advantages and disadvantages. Individual education is usually used at the beginning of treatment or in case of relapse, while group education has the advantage of promoting interpersonal communication and the exchange of ideas among equals, but it can also have drawbacks, as there may be resistance to change and lack of awareness of the disease [36]. In this study, despite having studies of both types, it was not possible to determine which of the two types of education is most effective for this type of patients. This analysis was not possible due to the heterogeneity among studies.

Third, we were unable to deeply analyze the intervention-effect relationship. The intensity of the intervention and compliance with the implementation protocol is considered important for education to be effective. Therefore, it would have been valuable to evaluate the quality of the intervention in addition to the quality of the articles. However, the articles reviewed lacked the necessary information to do so.

Fourth, studies were included in which educational interventions were explicitly described, whether they were the main treatment program or were part of it. However, it is possible that other articles address interventions in this population in which education has not been made explicit as part of the intervention.

Fifth, although education can improve knowledge, a direct impact on the behavior of patients is not necessarily realized. These changes may be exclusively due to education or to education and other related factors.

Sixth, the evidence that family involvement improves the effectiveness of educational interventions by facilitating the practical application and adherence of all its members to the proposed new behavioral models [37] especially in the case of children and adolescents [38]. In our work, we had both studies that included families and those that did not; however, it was not possible to determine if they play a relevant role, due to the methodological limitations previously mentioned.

We believe that future studies that aim to measure the effectiveness of educational interventions in young patients with feeding and eating disorders should not only evaluate the cognitive and behavioral benefits in terms of greater knowledge and better self-management but also examine the psychological aspects, biological and sociocultural development [39] related to personal well-being, identity development, socialization [40], family functioning and other dimensions that are affected by the appearance of this type of pathology [41].

Considering that existing evidence suggests that education improves different health parameters, it must be determined how and why the educational interventions examined in this review were effective in achieving their objectives. Only by obtaining a deeper understanding, can we generalize our results beyond current studies and provide constructive information for the design of educational programs aimed at this population. It is likely that the use of qualitative research methods is appropriate in this context.

The findings obtained in this work suggest that educational interventions can play an important role in the treatment of feeding and eating disorders, producing improvements beyond mere knowledge acquisition. This finding may be useful for professionals involved in the treatment and care of patients with FED’s. Especially relevant is the role played by mental health nurses, since both professionals and patients consider nursing interventions to be fundamental [42]. In the usual development of their functions, mental health nurses use educational interventions to provide patients with nutritional advice or instructions on how to replace pathological eating patterns with healthier ones [43]. Moreover, education in adolescents is identified by both patients and mental health nurses as an essential part of nursing care in people diagnosed with feeding and eating disorders [7].

Despite only 10 studies were analyzed due to the strict methodological rigor established by the PRISMA guidelines, a large number of studies were reviewed. This is the first review that addresses this concept in the adolescent population, offering a detailed description and analysis of the existing evidence, as well as illustrating its scarcity.

### Strengths and limits

One of the strengths of our systematic review is that to our knowledge, this is the first systematic review assessing the effect of educational interventions on the improvement of acquired knowledge, as well as on other variables, in the young population diagnosed with feeding and eating disorders.

Additional limitations include the inclusion of various types of studies with varying methodological quality and evidence level, the possible exclusion of relevant studies due to restrictive inclusion criteria, the limitation to only two languages, and the consideration that the observed effects may be influenced by factors other than the educational intervention. Additionally, due to the significant heterogeneity of the included studies and results, it was not possible to conduct a meta-analysis, thus limiting the results to a qualitative analysis.

## Conclusion

Health education has traditionally been considered a fundamental part of treatment in young patients diagnosed with feeding eating disorders. Despite this, in a preliminary search, empirical data on its efficacy were scarce and inconsistent. To confirm the significance or educational interventions, we conducted this systematic review on their impact on improving health outcomes in young people with eating disorders.

Our work allows us to conclude that educational interventions in adolescent  patients diagnosed with eating disorders seem to improve their level of knowledge and directly affect their health outcomes at different levels. Although an extensive literature search has been carried out and a large number of articles have been reviewed, it has not been possible to locate many works of high methodological quality. Further, the results of the studies obtained are heterogeneous, so future research will be necessary to confirm the results obtained.

### What is already known on this subject?

Feeding and Eating disorders are prevalent disorders among young people, and many treatments include educational interventions. However, there is limited evidence available on the effectiveness of these interventions in this group of patients.

### What does this study add?

There is an emerging body of evidence suggesting that educational interventions have an impact on both knowledge acquisition and various health-related parameters. However, this evidence is characterized by a low number of randomized controlled trials in this area. Our review highlights the need for future research to guide the development of effective educational interventions. The datasets used and/or analysed during the current study are available from the corresponding author on reasonable request.

### Supplementary Information

Below is the link to the electronic supplementary material.

Supplementary file1 (DOCX 13 KB)

Supplementary file2 (DOCX 33 KB)

## Data Availability

The datasets used and/or analysed during the current study are available from the corresponding author on reasonable request.
