# Supplementary material for: Efficacy of educational interventions in adolescent population with feeding and eating disorders: a systematic review
Source: Eat Weight Disord. 2023 Aug 22;28(1):69. doi: 10.1007/s40519-023-01594-9 (PMC10444681; doi:10.1007/s40519-023-01594-9)
Supplement: Supplementary file 1 — Supplementary file1 (DOCX 13 KB) [file 40519_2023_1594_MOESM1_ESM.docx]

Appendix 1: Search strategy (Pubmed):

| **Search** | **Query** |
| --- | --- |
| #1 | adolescence[Title/Abstract] |
| #2 | young[Title/Abstract] |
| #3 | youth[Title/Abstract] |
| #4 | teen[Title/Abstract] |
| #5 | teens[Title/Abstract] |
| #6 | kid[Title/Abstract] |
| #7 | kids[Title/Abstract] |
| #8 | child[Title/Abstract] |
| #9 | children[Title/Abstract] |
| #10 | teenager[Title/Abstract] |
| #11 | teenagers[Title/Abstract] |
| #12 | adolescent[MeSH Terms] |
| #13 | child[MeSH Terms] |
| #14 | infant[MeSH Terms] |
| #15 | (#1 OR #2 OR #3 OR #4 OR #5 OR #6 OR #7 OR #8 OR #9 OR #10 OR #11 OR #12 OR #13 OR 1#14) |
| #16 | (Feeding and Eating Disorders[MeSH Terms]) |
| #17 | feeding disorder*[Title/Abstract] |
| #18 | eating disorder*[Title/Abstract] |
| #19 | appetite disorder*[Title/Abstract] |
| #20 | anorexia nervosa[Title/Abstract] |
| #21 | bulimia nervosa[Title/Abstract] |
| #22 | PICA[Title/Abstract] |
| #23 | Avoidant Restrictive Food Intake Disorder[Title/Abstract] |
| #24 | ARFID[Title/Abstract] |
| #25 | rumination disorder*[Title/Abstract] |
| #26 | Binge-Eating Disorder[Title/Abstract] |
| #27 | (#16 OR #17 OR #18 OR #19 OR #20 OR #21 OR #22 OR #23 OR #24 OR #25 OR #26) |
| #28 | Education[MeSH Terms] |
| #29 | Health education[MeSH Terms] |
| #30 | education[Title/Abstract] |
| #31 | educational[Title/Abstract] |
| #32 | teach[Title/Abstract] |
| #33 | teaching[Title/Abstract] |
| #34 | train[Title/Abstract] |
| #35 | training[Title/Abstract] |
| #36 | learn[Title/Abstract] |
| #37 | learning[Title/Abstract] |
| #38 | (#28 OR #29 OR #30 OR #31 OR #32 OR #33 OR #34 OR #35 OR #36 OR #37) |
| #39 | (#15 AND #27 AND #38) |
